# Serum HMGB1 as a diagnostic biomarker and mediator of childhood trauma in adolescent depression

**DOI:** 10.3389/fpsyt.2025.1584320

**Published:** 2025-06-10

**Authors:** Jingyue Xiang, Yiqun Qin, Ruhong Jiang, Xiaolan Wang, Yang Zhou, Jia Liu, Li Kuang

**Affiliations:** ^1^ Department of Psychiatry, The First Affiliated Hospital of Chongqing Medical University, Chongqing, China; ^2^ Psychiatric Center, The First Affiliated Hospital of Chongqing Medical University, Chongqing, China; ^3^ First Clinical Medical College, Chongqing Medical University, Chongqing, China; ^4^ Mental Health Center, University-Town Hospital of Chongqing Medical University, Chongqing, China

**Keywords:** depression, HMGB1, biomarkers, inflammation, adolescents

## Abstract

**Background:**

Adolescent depression is a global health challenge with increasing rates and long-term impacts on development. Current diagnostics lack objective biomarkers, relying on subjective assessments. Neuroinflammation, particularly High mobility group box 1 (HMGB1), a systemic inflammation mediator, is implicated in adult depression but not well-studied in adolescents. Childhood trauma, a risk factor for neuroinflammatory dysregulation, has been linked to increased inflammatory markers but not specifically to HMGB1. This study explores serum HMGB1 as a diagnostic biomarker for adolescent depression and its role in exacerbating symptoms through childhood trauma.

**Methods:**

160 participants, including 80 depressive adolescents and 80 healthy controls, were enrolled. The depressive symptoms of depressive adolescents were evaluated with clinical scale. Serum HMGB1 levels were measured by enzyme-linked immunosorbent assay (ELISA). Correlation analysis, multiple linear regression, and mediation effect analysis were utilized to examine the relationship between serum HMGB1 levels and depressive symptoms severity.

**Results:**

Compared with the control group, serum HMGB1 levels (t = -18.48, *P <*0.001) was increased in depressive adolescents. Correlation analysis showed that serum HMGB1 levels in depressive adolescents were positively correlated with 17-item Hamilton Depression Rating Scale (HAMD-17) scores and Childhood Trauma Questionnaire (CTQ) scores. Multiple linear regression analysis showed that serum HMGB1 levels can independently predict HAMD-17 scores for depressive adolescents. HMGB1 demonstrated high diagnostic accuracy (AUC = 0.984) and significantly mediated depressive symptoms through childhood trauma (indirect effect = 0.0028, 95% CI: 0.0008-0.0058).

**Conclusion:**

Serum HMGB1 levels are potential markers of depression, and childhood trauma partially mediates the relationship between HMGB1 and depressive symptoms severity.

## 1 Introduction

Depression is a prevalent mental disorder among children and adolescents, marked by elevated recurrence, disability, and suicide risk, posing a substantial burden on families and society (1).The pooled one-year prevalence of depression among adolescents is 8%, and the lifetime prevalence is 19% (2). Globally, depression is one of the leading contributor of suicide among adolescents, accounting for a substantial proportion of mortality in this age group (3). Adolescent depression frequently manifests atypical clinical symptoms and unclear pathophysiological mechanisms, resulting in suboptimal treatment outcomes (4). An in-depth exploration of its biological mechanisms can optimize the accuracy of early diagnosis, enhance treatment efficacy, and uncover the biological underpinnings of its atypical clinical manifestations.

High mobility group box 1 (HMGB1), a non-histone nuclear protein, is a pivotal molecule with diverse functions (5). It plays a crucial role in maintaining nucleosome structure, regulating gene transcription, and contributing to chromatin stability and DNA recombination processes (6). A core functional mechanism of HMGB1 is mediated by its interaction with Toll-like receptor 4 (TLR4) and receptor for advanced glycation end products (RAGE), which subsequently activates downstream signaling pathways, triggering inflammatory and oxidative stress responses (7). Notably, HMGB1, classified as a damage-associated molecular pattern (DAMP), serves as an alarm signal for damage or stress in the body and is recognized by the immune system at the early stage of inflammation, initiating an immune response. Concurrently, HMGB1 exhibits significant sustained release in the later phase of acute inflammation, interacting with HMGB1-induced proinflammatory cytokines to form a positive feedback loop, continuously amplifying the inflammatory response (8). HMGB1 exerts a pivotal function primarily through the TLR4/NF-κB signaling pathway, ultimately leads to the secretion of proinflammatory cytokines, including TNF-α and IL-6, as reported in studies (9–11). In addition to its roles in chronic diseases, HMGB1 also plays multiple roles in immune responses. It modulates immune reactions by promoting the polarization of Th2 and Th17 cells and the production of related factors (12). Furthermore, HMGB1 modulates the functions of Treg and Th17 cells through its interaction with RAGE, facilitating cell migration, mediating the production of IL-18, and enhancing the adhesion capacity of neutrophils (13). Notably, the oxidative modification of HMGB1 significantly alters its functions. Upon oxidized, HMGB1 forms oxidized HMGB1 (ox-HMGB1), which loses its original biological activity and is unable to bind to receptors, thereby diminishing its proinflammatory effects (14).

Recent research has increasingly explored HMGB1’s role in psychiatric disorders. Elevated HMGB1 concentrations have been observed in the blood plasma of individuals diagnosed with autism spectrum disorder. In rodent models of autism, HMGB1 exacerbates vascular permeability and leukocyte infiltration via the HMGB1/RAGE/TLR4 axis, leading to persistent neuroinflammation (15). Schizophrenia patients display higher serum HMGB1 levels than controls. There is no statistically significant variation in biomarker levels between acute and remission phases of schizophrenia, and HMGB1 levels in remission-phase patients correlate with the severity of symptoms assessed by psychiatric scales (16). Preclinical studies have demonstrated that injecting recombinant HMGB1 (rHMGB1) into the lateral ventricles of neonatal mice can induce depression-like behaviors, while anti-HMGB1 antibody treatment suppresses microglial activation and ameliorate these behaviors (17). A clinical investigation has revealed that elevated serum HMGB1 levels correlate with the severity of depression in adult patients with depression (18). HMGB1’s role in depression pathophysiology is underscored by its release kinetics. Chronic stress-induced neuronal damage triggers early passive HMGB-1 leakage, initiating microglial activation via TLR4/RAGE signaling. Subsequently, sustained HMGB-1 secretion from activated glia perpetuates neuroinflammation through cytokine feedback loops, a mechanism aligned with depression’s chronicity. Unlike short-lived cytokines, such as TNF-α, HMGB1’s prolonged detectability enhances its clinical relevance (19). Furthermore, HMGB1 directly compromises blood-brain barrier integrity, facilitating peripheral-central immune communication (20, 21). HMGB1 has a unique advantage over cytokines requiring secondary mediators for central nervous system effects. As a key mediator of neuroinflammation, HMGB1 has emerged as a promising candidate in adult depression, yet its diagnostic utility and mechanistic role in adolescents remain unexplored.

Multiple studies have demonstrated a significant correlation between childhood trauma and the risk of adult depression. A meta-analysis comprising 184 studies revealed that found that individuals who experienced childhood trauma had a substantially increased likelihood of developing depression in adulthood, with risk estimates ranging from 2.66 to 3.73 times higher (22). Another study highlighted that childhood trauma is a crucial correlate of suicidal ideation among depression patients (23). Childhood trauma influences mental health in adulthood through various biological mechanisms, including HPA axis dysregulation, epigenetic modifications, and neuroinflammation, which increase depression vulnerability (24). A meta-analysis examining the relationship between childhood trauma and adult inflammation identified TNF-α as the most strongly correlated inflammatory marker, followed by IL-6 and CRP. The analysis indicated that childhood trauma intensifies adult inflammatory responses, suggesting a significant and enduring impact of childhood traumatic events on inflammatory processes (25). A study revealed that depressed patients who had experienced childhood trauma exhibited significantly elevated IL-6 levels, which correlated with childhood trauma history questionnaire scores. This suggests that childhood trauma may increase the risk of depression by influencing the expression of inflammatory markers (26). Childhood trauma, a significant risk factor for depression, may interact with inflammatory pathways to exacerbate symptom severity. However, no prior study has examined whether HMGB1 influences depressive symptoms through trauma-associated mechanisms.

Here, we hypothesize that serum HMGB1 not only discriminates depressed adolescents from healthy controls but also mediates symptom severity via childhood trauma. By integrating biomarker discovery with mechanistic exploration, this study aims to advance both etiological understanding and clinical translation for adolescent depression.

## 2 Materials and methods

### 2.1 Subjects and clinic assessment

This research was conducted as a cross-sectional investigation, enrolling 80 adolescent individuals diagnosed with depression from the First Affiliated Hospital of Chongqing Medical University. *A priori* sample size calculation was performed using GPower 3.1. Based on preliminary HMGB1 data (depression group: 2725.09 ± 395.83 pg/ml; control group: 1462.53 ± 469.68 pg/ml), he calculated effect size (Cohen’s d = 2.91) indicated an extremely large effect. To prevent overfitting caused by potential biases in the pilot data, we conservatively assumed a moderate effect size (Cohen’s d = 0.5). With α=0.05 and power=0.8, the required sample size was 128 (64 per group). Anticipating 20% data loss or outliers, we enrolled 160 participants (80 per group). Anticipating potential data variability and data loss or outliers, we enrolled 160 participants (80 per group). The inclusion criteria were established in accordance with the guidelines specified in the fifth edition of the Diagnostic and Statistical Manual of Mental Disorders (DSM-5), with diagnoses being confirmed by two experienced, senior clinical practitioners. None of the patients had received any antidepressant treatment. Inclusion criteria (1): aged between 12 and 17 years, Han nationality (2); 17 items of Hamilton Depression Scale score ≥17. An additional 80 age- and gender-matched healthy adolescents served as the control group. Exclusion criteria (applicable to both patients and controls) (1): Psychiatric disease history or family history (2); history of brain organic diseases or severe physical illnesses (3); psychoactive substance abuse (4); acute or chronic infectious diseases or communicable diseases with infectious symptoms in the past 2 weeks (5); use of anti-inflammatory, immunomodulatory, or hormonal drugs in the month prior to the study. The Ethics Committee of Chongqing Medical University’s First Affiliated Hospital gave its approval for this investigation (2021–546), and all subjects and their guardians signed informed consent forms.

### 2.2 Depressive symptoms

17-item Hamilton Depression Scale (HAMD-17) is employed to assess depressive symptoms in patients, consisting of 17 items (27). Among them, eight items are scored on a scale ranging from 0 to 2, whereas nine items are evaluated on a scale spanning from 0 to 4. A higher total score indicates more severe depressive symptoms, serving as the gold standard for clinically assessing the severity of depression. The HAMD-17 scoring criteria are typically used to define depression severity as follows: a total score of ≥24 indicates severe depression, 17–24 moderate depression, 8–17 mild depression, and ≤7 no depressive symptoms. In this study, depressive adolescents were required to have a HAMD-17 score of ≥17 (28).

### 2.3 Assessment of childhood trauma

The Childhood Trauma Questionnaire (CTQ) is employed for assessment purposes, consisting of 28 items that address emotional abuse, physical maltreatment, sexual assault, emotional neglect, and physical disregard. A Likert-type scale with five points, spanning from “Never” to “Almost Always,” is employed, with increased scores indicating a higher level of severity in traumatic experiences (29).

### 2.4 Serum HMGB1 levels

Blood samples were collected from subjects in a fasting state, with 5 milliliters of peripheral venous blood drawn into tubes without anticoagulants. Natural coagulation of the blood occurred at room temperature for a duration of 20 minutes, subsequently subjected to centrifugation at 3000 revolutions per minute to isolate the serum component. The isolated serum was preserved at -80°C, awaiting further analytical procedures. The serum HMGB1 levels were determined using the human HMGB1 ELISA kit provided (Jiubang Biotechnology, Fujian, China), observing the manufacturer’s directions.

### 2.5 Statistical analysis

Continuous variables were tested for normal distribution using the Shapiro-Wilk test, and based on the outcome, we utilized the Mean ± SD to describe their characteristics. Data conforming to normality were analyzed with independent t-tests and Pearson correlation, while non-normally distributed variables were analyzed using Mann-Whitney U test and Spearman’s correlation. For categorical data, we presented the results as n (%), and employed chi-square tests to compare differences between groups. To ascertain whether HMGB1 levels can function as a standalone predictor for depression, we utilized correlation analysis alongside multiple linear regression models. Receiver Operating Characteristic (ROC) curve analysis determined the diagnostic accuracy of HMGB1. Mediation analysis was conducted using PROCESS macro (Model 4) with 5,000 bootstrap resamples. All analyses were performed in SPSS 26.0 with statistical significance set at *P* < 0.05.

## 3 Results

### 3.1 Sociodemographic and clinical feature

This research enrolled a collective of 160 participants, comprising 80 adolescents diagnosed with depression and an additional 80 healthy controls. Significant differences were found between the two groups in HMGB1 levels, HAMD-17 scores, and CTQ scores, as detailed in **Table 1** (*P* < 0.05). However, no significant differences were observed in age, gender, or BMI (*P* > 0.05).

**Table 1:** Sociodemographic and clinical features of participants.

Variables	Control (n = 80)	Depression (n = 80)	t/χ²	*P*
Age, years, Mean ± SD	15.26 ± 0.74	15.29 ± 1.12	-0.17	0.868
Gender, n (%)			1.16	0.281
Male	24 (30.00)	18 (22.50)		
Female	56 (70.00)	62 (77.50)		
BMI, Mean ± SD	20.08 ± 2.77	20.77 ± 3.76	-1.32	0.188
HMAD-17, Mean ± SD	1.15 ± 1.48	23.46 ± 4.20	-44.79	<0.001
CTQ, Mean ± SD	35.73 ± 9.85	48.83 ± 12.95	-7.20	<0.001
HMGB1, pg/ml, Mean ± SD	1519.78 ± 365.69	2587.05 ± 364.80	-18.48	<0.001

BMI, body mass index; HAMD-17, 17-item Hamilton Depression Scale; CTQ, Childhood Trauma Questionnaire; SD, standard deviation.

### 3.2 Correlation of depressive symptom severity with serum HMGB1 levels in adolescents

**Figure 1:**
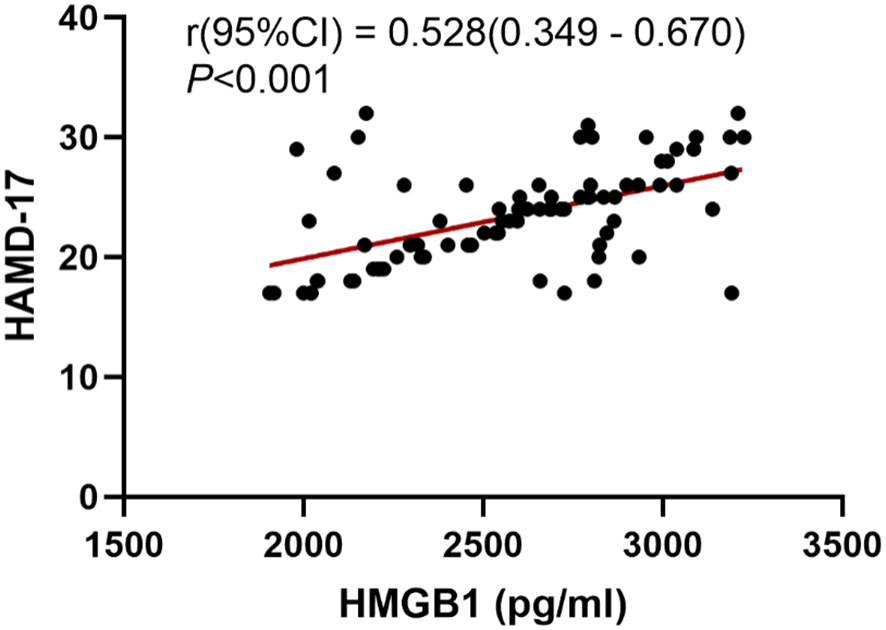
Correlation between depression severity and serum HMGB1 levels.

**Figure 2:**
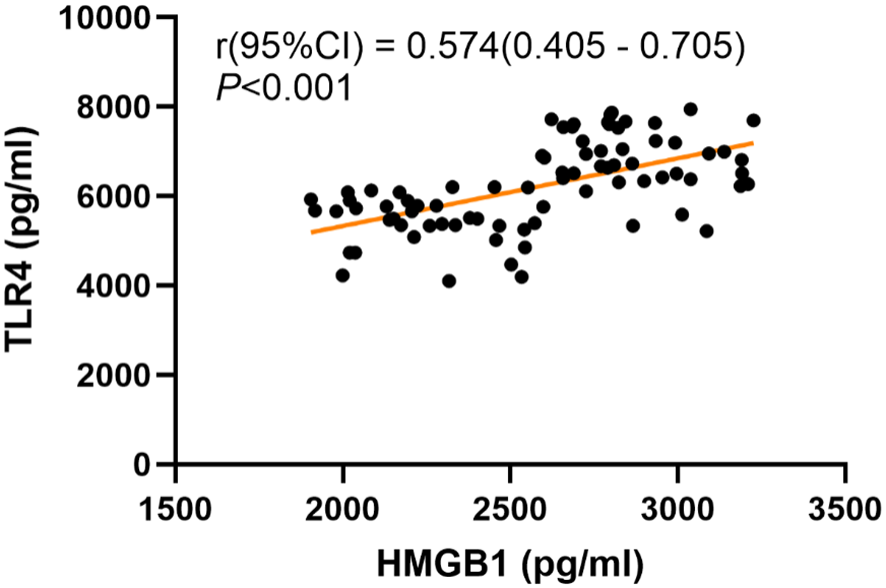
Correlation between serum HMGB1 levels and TLR4 levels.

We conducted further analysis to examine the correlation between the severity of depressive symptoms and serum HMGB1 levels. The results of the Pearson correlation analysis are presented in **Figure 1**. A significant positive correlation was observed between serum levels of HMGB1 and HAMD-17 scores (r = 0.528, *P* < 0.001). To explore the relationship between HMGB1 and its canonical receptor TLR4, we measured serum TLR4 levels. Serum HMGB1 levels showed a significant positive correlation with TLR4 levels in the depression group (r=0.574, P<0.001), suggesting HMGB1-TLR4 pathway activation, as shown in **Figure 2**.

Multivariate linear regression analyses were conducted, using HAMD-17 scores as the dependent variable and serum HMGB1 concentrations as the independent variable, with adjustments for confounding variables such as sex, age, and BMI. The results demonstrated that HMGB1 remained an independent predictor of HAMD-17 scores, as shown in **Table 2**.

**Table 2:** Multiple linear regression analysis between HAMD-17 and HMGB1 levels.

Variables	Model1		Model2	
	*β* (95%CI)	*P*	*β* (95%CI)	*P*
HMGB1	0.01 (0.01 ~ 0.01)	<0.001	0.01 (0.01 ~ 0.01)	<0.001

CI, confidence interval.

Model1: Crude.

Model2: Adjust, Gender, age, BMI.

### 3.3 Assessment of the diagnostic performance of HMGB1

**Figure 3:**
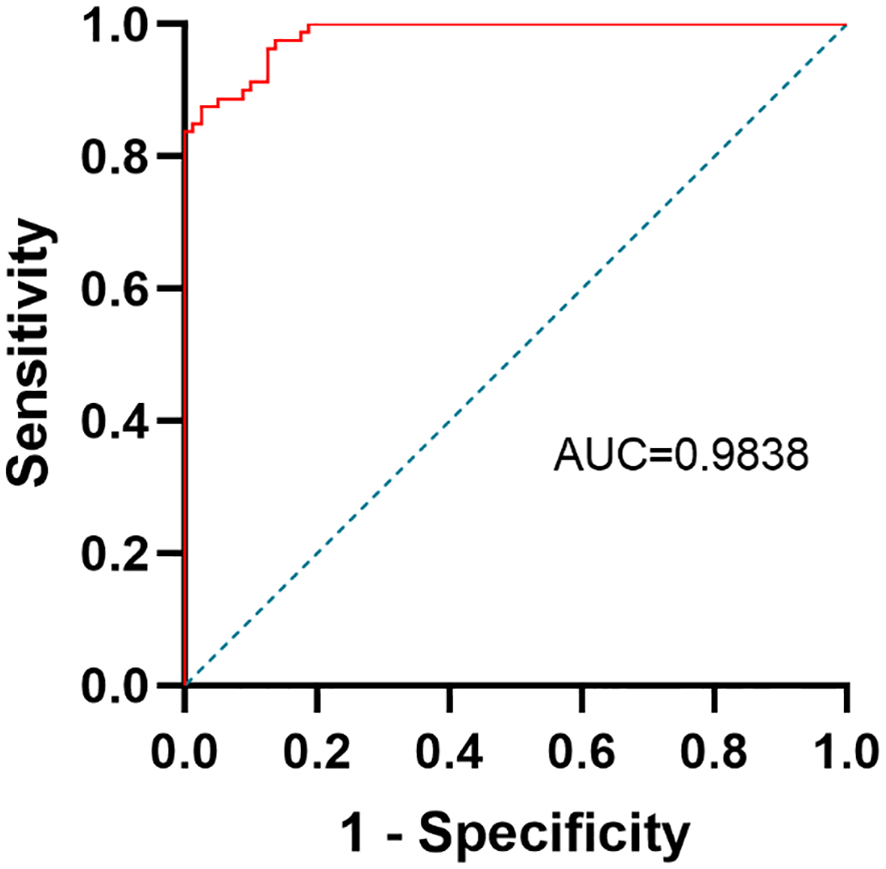
ROC curve of serum HMGB1 for discriminating depressive adolescents from healthy controls.

The differential power between depressive adolescents and healthy adolescents was predicted by a ROC curve analysis. HMGB1 was selected as a potential predictor since it was independently linked to the severity of depressive symptoms and showed a significant difference between the depression and controls. The ROC curve is shown in **Figure 3**. The AUC, specificity, sensitivity and cut-off value of HMGB1 for identifying adolescent depression and controls was 0.9838, 97.5%, and 87.5%, 2117 pg/ml.

### 3.4 Mediating effects of childhood trauma in HMGB1 level and depression

**Figure 4:**
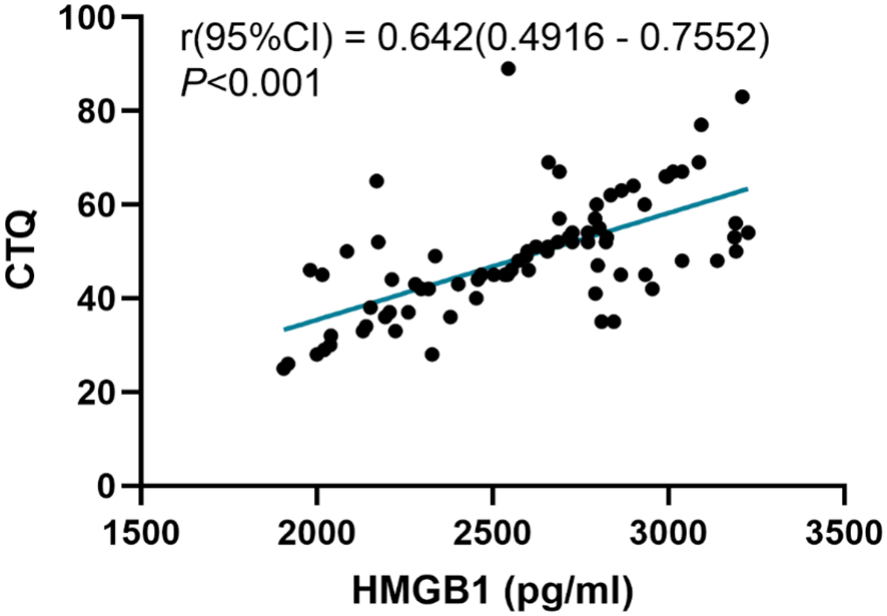
Correlation between childhood trauma and serum HMGB1 level.

We analyzed the correlations between the childhood trauma and serum HMGB1 levels. The Pearson correlation analysis is shown in **Figure 4**. There was a positive correlation between the CTQ scores and the HMGB1 levels (r = 0.642, *P* < 0.001).

Mediation analysis demonstrated a significant total effect of HMGB1 on HAMD scores (β=0.0061, 95% CI: 0.0039–0.0083, P<0.001). After controlling for CTQ scores, HMGB1 still had a direct positive impact on HAMD scores (β=0.0033, 95% CI: 0.0005–0.0060, *P*=0.020), but the effect size decreased by 45.9%. Further analysis showed a significant indirect effect of HMGB1 through CTQ scores (β=0.0028, 95% CI: 0.0008–0.0058), contributing 45.9% of the overall effect, suggesting that childhood trauma partially mediates the relationship between HMGB1 and depressive symptoms, as shown in **Table 3**.

**Table 3:** Mediating effects of childhood trauma in HMGB1 levels and depression.

Effect Type	Effect	SE	95% *CI*	*P*
Total Effect (HMGB1→HMAD-17)	0.0061	0.0011	[0.0039, 0.0083]	< 0.001
Direct Effect (HMGB1→HMAD-17)	0.0033	0.0014	[0.0005, 0.0060]	0.0195
Indirect Effect (HMGB1→CTQ→HMAD-17)	0.0028	0.0012	[0.0008, 0.0058]	–

HAMD-17, 17-item Hamilton Depression Scale; CTQ, Childhood Trauma Questionnaire; SE, standard error; CI, confidence interval.

## 4 Discussion

In this study, we found that adolescents diagnosed with depression exhibited notably higher levels of serum HMGB1 compared to healthy counterparts, with a clear positive correlation between HMGB1 levels and depressive symptom severity, further confirming serum HMGB1 as a highly sensitive and specific diagnostic biomarker for adolescent depression. Additionally, we found that childhood trauma partially mediates the association between HMGB1 and depressive severity, uncovering a novel neuroinflammatory-psychosocial pathway. This study is the first to elucidate how HMGB1 indirectly influences adolescent depressive symptoms through childhood trauma. Our findings supported the inflammatory links between childhood trauma and psychopathology (30), indicating that HMGB1 exacerbates depression both directly through neuroinflammatory responses and indirectly by increasing individual susceptibility to the effects of childhood trauma.

In our study of adolescent depression, we found elevated levels of HMGB1, consistent with previous clinical studies in adult depression which reported significantly higher serum HMGB1 levels compared to controls (31). Further analysis revealed that the AUC value for HMGB1 was higher in adolescent depression patients, potentially indicating more sensitive neuroinflammatory responses in this population. Considering that adolescence is a peak period for depression onset and neuroinflammatory responses may be more pronounced at this stage (32, 33), elevated HMGB1 levels in adolescent depression may be more evident. While our exclusion criteria minimized confounding from common HMGB1-elevating conditions, some limitations remain. First, subclinical inflammation such as undiagnosed periodontal disease may have contributed to HMGB1 variability (34). Second, HMGB1 elevation can occur in non-inflammatory conditions including traumatic brain injury, and these conditions were not systematically screened in this study. However, the lack of documented severe physical illness in our cohort suggests that such confounders are unlikely to substantially affect the primary findings (35). This study is the first to validate the diagnostic efficacy of HMGB1 in adolescents, addressing a gap in age-specific data. Given the complexity of diagnosing and treating adolescent depression and the potential inaccuracy of traditional symptomatic assessments, detecting HMGB1 as a biomarker holds significant importance for providing a more objective diagnostic basis.

Previous research has demonstrated a significant synergistic effect between inflammation and trauma. Specifically, Danese et al. previously elucidated the crucial role of CRP in mediating the association between trauma and depression (36), yet the potential mediating role of HMGB1 in this process remains inadequately explored. The hippocampus, a brain region for memory formation and emotional regulation (37), exhibits a close correlation between HMGB1 expression levels and the processes of neuroinflammation and neurodegeneration. In models of hypoxic-ischemic brain damage, HMGB1 induces the release of pro-inflammatory cytokines by activating astrocytes and microglia, leading to neuronal damage (38). Furthermore, HMGB1 can also induce inflammatory responses in the hippocampus via the TLR4-NF-κB signaling pathway (39). Our correlation analysis revealed a strong association between HMGB1 and TLR4 in depressed adolescents, aligning with preclinical evidence that HMGB1-TLR4 signaling drives neuroinflammation via NF-κB and NLRP3 activation. Our study found that HMGB1 partially mediates the relationship between depressive symptoms and childhood trauma, as assessed by CTQ scores. The mechanism may involve childhood trauma activating inflammatory pathways, promoting the expression and release of HMGB1. HMGB1 binds to the TLR4 receptor on the surface of microglia and astrocytes through its B-box domain, with the strongest activity in the disulfide form, activating the MyD88-dependent signaling pathway. The activation of TLR4 triggers the phosphorylation of IκBα mediated by the IKK complex and subsequent proteasomal degradation, promoting the translocation of the NF-κB p65/p50 heterodimer from the cytoplasm into the nucleus. Nuclear NF-κB drives the neuroinflammatory cascade by binding to the gene promoter regions of pro-inflammatory cytokines such as IL-1β, TNF-α, IL-6 (40). These cytokines further exacerbating inflammatory responses in the hippocampus and enhancing its sensitivity to trauma-related cues, thereby facilitating the consolidation of traumatic memories. Previous studies demonstrated that in patients with depression comorbid with temporal lobe epilepsy, cytoplasmic translocation of HMGB1 in hippocampal CA region neurons was significantly increased. The cytoplasmic accumulation of HMGB1 in this area was suggested to trigger localized inflammation via the TLR4/NF-κB pathway, which subsequently disrupted synaptic plasticity and neurotransmitter homeostasis. Furthermore, inflammatory factors exacerbated hippocampal damage through blood-brain barrier leakage, ultimately forming a comorbid mechanism linking epilepsy and depression (41, 42). HMGB1 may influence depression through pathways other than those mediated by childhood trauma. A systematic review of seven studies suggested that stress-induced inflammation mediated by HMGB1 regulates the dopamine pathway within corticostriatal neurocircuitry, thereby potentially influencing motivational deficits (43).

Recent research has demonstrated that epigenetic variations induced by childhood trauma exhibit not only significant long-term persistence but also the potential for transgenerational transmission, revealing their extensive impact on gene expression and potential effects on offspring’s physiological health (44–46). Specifically, childhood trauma can elicit marked alterations in DNA methylation patterns, finely regulating gene transcriptional activity. Serena’s research found that after childhood trauma, the promoter region of the glucocorticoid receptor gene (NR3C1) exhibited a characteristic of high methylation, significantly correlating with an enhanced sensitivity to stress responses and a greater likelihood of mental illnesses (47). Additionally, research has indicated that childhood trauma can cause specific changes in the methylation status of critical genes such as FKBP5 and KITLG, which are intimately associated with individual stress response mechanisms and the deterioration of mental health status (48–50). Notably, HMGB1, a key protein in inflammatory response and immune regulatory networks, may undergo sustained overexpression at its genomic loci due to DNA methylation modifications induced by childhood trauma, thereby exerting a profound influence on the dynamics of inflammatory responses and the immune functional status of the organism.

Recent scientific studies have demonstrated that anti-HMGB1 monoclonal antibodies effectively suppress microglial activation and reduce pro-inflammatory cytokine secretion, significantly decreasing inflammatory levels in the brain (51). Glycyrrhizin, as an HMGB1 inhibitor, has been approved by the FDA for clinical application (52). In depression models such as neuropathic pain, Glycyrrhizin can inhibit microglial activation, thereby improving depressive-like behaviors (53). For patients with high HMGB1 levels, psychological interventions such as Eye Movement Desensitization and Reprocessing (EMDR) may be more effective. EMDR alleviates depressive symptoms by activating the brain’s desensitization process and reducing the impact of traumatic memories (54). Recent clinical trials underscore the therapeutic potential of modulating inflammatory pathways in depression. Aldossary demonstrated that high-dose atorvastatin, as an add-on therapy, significantly improved depressive symptoms by suppressing the AMPK/NLRP3 inflammasome and IL-6/STAT3 axes pathways (55). Similarly, El-Haggar reported that pentoxifylline, a phosphodiesterase inhibitor, enhanced antidepressant efficacy via TNF-α inhibition (56). While these studies target downstream cytokine networks, our focus on HMGB1, a master regulator of sterile inflammation, offers a upstream intervention point. This suggests that early HMGB1 blockade could prevent maladaptive neuroinflammation. Furthermore, combining HMGB1 inhibitors such as glycyrrhizin with cytokine - targeted agents such as pentoxifylline may yield synergistic effects, a hypothesis that merits future clinical trials. These research findings present new perspectives and approaches to treating adolescent depression, indicating that by regulating the expression level of HMGB1, it may be possible to significantly improve depressive symptoms in patients, bringing new breakthroughs to clinical treatment of depression.

The diagnosis of depression primarily relies on patient self-report or clinical interviews conducted by physicians, lacking objective biomarkers for assistance. Despite subjective assessment being the core method, it entails risks of diagnostic heterogeneity, misdiagnosis, and missed diagnosis. Serum HMGB1 levels can be objectively measured through standardized tests and exhibit a high correlation with the chronic inflammatory state of depression, demonstrating potential as an auxiliary indicator. However, HMGB1’s clinical integration requires further validation through multicenter studies and cost-benefit analyses to justify routine use.

Our study has several limitations. Firstly, even though it has been shown that HMGB1 is linked to depressed symptoms and that childhood trauma mediates these symptoms, the cross-sectional design cannot establish causality and temporal sequence. Secondly, to control for confounding factors, patients undergoing antidepressant treatment or with other psychiatric disorders were excluded, enhancing internal validity but limiting the generalizability of the results. Thirdly, although our sample size provided adequate power for detecting large HMGB1 effects (Cohen’s d=0.8), it may have been underpowered to detect smaller but clinically meaningful effects. Future multi-center studies with larger cohorts are needed to validate these findings. Our cohort was exclusively recruited from the Han Chinese ethnic group in a single province. Given known ethnic and regional variations in immune profiles, external validation in diverse populations is critical. Lastly, the focus solely on HMGB1 without concurrent assessment of other inflammatory markers hampers the evaluation of its specificity within the inflammatory network.

Future research should focus on several key areas. Firstly, exploring the relationship between dynamic changes in HMGB1 and depressive course as well as treatment response through longitudinal cohort studies. Secondly, revealing the multidimensional regulatory network of HMGB1 by utilizing multi-omics integration analysis, combining genomic, epigenetic, and neuroimaging data. Thirdly, future research directions should emphasize multicenter randomized controlled trials investigating targeted anti-HMGB1 interventions, including glycyrrhizin derivatives, with particular attention to adolescent cohorts stratified according to baseline HMGB1 concentrations. Therapeutic agents should be prioritized based on their ability to penetrate the blood-brain barrier while demonstrating minimal immunosuppressive effects. For instance, the HMGB1 A-box fragment represents a promising candidate due to its capacity to neutralize extracellular HMGB1 signaling without broadly suppressing immune function (57), thereby preserving developmental immunity in adolescents. Fourthly, dissecting the complex causal chain among HMGB1, CTQ, and HAMD-17 through cross-species validation, particularly in animal models such as maternal separation models.

Overall, our study demonstrates that HMGB1 serves as an efficient diagnostic biomarker for adolescent depression, with part of its effects mediated by childhood trauma, supporting a bio-psychosocial integrated intervention strategy. In conclusion, this study positions HMGB1 at the intersection of neuroinflammation and psychosocial adversity in adolescent depression. By bridging biomarker discovery with mechanistic elucidation, our findings advocate for dual-pathway interventions targeting both inflammatory dysregulation and trauma-associated vulnerabilities.

## Data Availability

The raw data supporting the conclusions of this article will be made available by the authors, without undue reservation.
